# Magnetic resonance-guided high-intensity focused ultrasound combined with radiotherapy for palliation of head and neck cancer—a pilot study

**DOI:** 10.1186/s40349-016-0055-x

**Published:** 2016-04-02

**Authors:** Justin Lee, Georges Farha, Ian Poon, Irene Karam, Kevin Higgins, Samuel Pichardo, Kullervo Hynynen, Danny Enepekides

**Affiliations:** Sunnybrook Health Sciences Centre, Department of Radiation Oncology, University of Toronto, Toronto, Canada; Sunnybrook Health Sciences Centre, Department of Head and Neck Surgery, University of Toronto, Toronto, Canada; Thunder Bay Regional Research Institute, Department of Physics, Lakehead University, Thunder Bay, Canada; Sunnybrook Health Sciences Centre, Department of Medical Physics, University of Toronto, Toronto, Canada

## Abstract

**Background:**

Radiotherapy is a critical component of the multidisciplinary management of cancers of the head and neck. It may comprise the primary curative treatment modality or is used in an adjuvant setting to improve local control and survival by preventing seeding and reseeding of distant metastases from persistent reservoirs of locoregional disease. Although considerable advances have been made recently in the fields of radiotherapy, systemic treatment and surgery for head and neck tumours, locoregional recurrence rates remain high and treatment side effects may have severe impact on patients’ quality of life.

Magnetic resonance-guided high-intensity focused ultrasound (MRg-HIFU) is a novel technique in the treatment of cancer that has the potential to improve tumour cure rates and decrease treatment-related toxicity. Clinical applications of HIFU are being used increasingly for the treatment of several tumour sites, for example uterine leiomyomas and prostate cancer.

**Methods/Design:**

The pilot study presented here is an initial step toward utilizing MRg-HIFU for head and neck cancer treatment. The rationale for novel treatment options in head and neck cancer is reviewed as well as emerging evidence that support the increasing clinical utilization of MRg-HIFU.

**Discussion:**

This pilot study aims to assess safety, toxicity and feasibility of MRg-HIFU treatments to the head and neck region and to evaluate changes caused by MRg-HIFU within the treated tumour regions based on post-treatment MRI.

## Background

### Head and neck cancer

Cancer of the head and neck is the sixth most common form of cancer diagnosed in the world; increasing incidence of oropharyngeal cancers has been reported in some regions which is likely linked with human papillomavirus-associated tumours [1]. Approximately 75 % of patients with cancer of the pharynx present with locally advanced or metastatic disease [2]. For patients with locally advanced disease, standard treatment typically includes a 7-week course of chemoradiotherapy alone or extensive surgery followed by post-operative chemoradiotherapy. These curative treatments can cause potentially severe side effects to the organs of the head and neck responsible for voice, speech, swallowing, taste and neurologic functions [3, 4].

The most common cause of initial treatment failure in patients with locally advanced head and neck cancer is recurrence at the primary site or in the lymph nodes of the neck [5, 6]. Despite combined modality treatments, 20–55 % of patients with locally advanced head and neck cancer will develop locoregional recurrence with overall survival of approximately 40–60 % [6–8]. Primary salvage treatments include re-irradiation and/or surgery. Unfortunately, those treatments are often limited by previous therapy, patient co-morbidities or the presence of distant metastasis. Median survival of patients with recurrent, metastatic disease is 3–6 months [9].

For patients with incurable disease due to recurrence, metastasis or severe medical co-morbidites, symptoms such as neck pain, dysphagia and respiratory difficulties affect patients’ quality of life. Intermediate doses of palliative radiotherapy have demonstrated response rates of approximately 50–70 % and improvements in quality of life but may entail more than 5 weeks of daily treatment and radiation-induced toxicities [3, 10, 11]. There remains a need for therapeutic strategies which can improve locoregional control and provide symptom relief while limiting treatment duration and side effects.

### MR-guided HIFU: clinical applications

Magnetic resonance-guided high-intensity focused ultrasound (MRg-HIFU) is a non-invasive, outpatient modality being investigated for the treatment of cancer. In MRg-HIFU, a specially designed transducer is used to focus a beam of ultrasound energy into a small volume at a specific target site in the body. The focused beam produces therapeutic heating (55–90 °C for 20–30 s) in the target field causing protein denaturation and cell damage resulting in tissue ablation. The tissue immediately adjacent to the target is warmed to a lower temperature which does not cause tissue ablation. Magnetic resonance (MR) imaging will be used both to focus the ultrasound beam on the target field in the neck (the metastatic lymph node or tumour mass—containing nerves and tumour vasculature) and to perform real-time thermal mapping in order to limit the ablative effects on the designated target and preserve healthy tissue.

Clinical applications of HIFU (which may use MR or other image guidance) are being used increasingly for the treatment of uterine leiomyomas and prostate cancer [12–15]. A recent prospective development study of 42 patients utilized MRI mapping and trans-rectal HIFU ablation of prostate tumours while sparing the normal gland tissue. HIFU treatment was associated with low rates of side effects and good early clinical control [15]. Early clinical studies have also explored HIFU techniques for the treatment of a wide array of solid tumour locations including the liver, kidney, breast, pancreas, bone and brain [16]. Current and potential intracranial applications include functional neurosurgery, relief of neuropathic pain, tumour ablation, drug delivery and thrombolysis [17]. Novel studies underway include a randomized, placebo-controlled phase III trial using MRg-HIFU to treat essential tremor and disruption of the blood-brain barrier to enhance drug delivery [17, 18]. Treatment of bone lesions including both benign and malignant tumours has been an active area of investigation. The results of a recently completed phase III trial demonstrate the utility of MRg-HIFU as a treatment option for patients with painful bone metastases [19, 20].

To our knowledge, at the time of writing this protocol, there are no prospective clinical data reported on the use of MRg-HIFU or other HIFU techniques in the setting of head and neck cancer. In vivo experiments of MRg-HIFU in a head and neck cancer mouse model demonstrated evidence of tumour de-vascularization, apoptosis and necrosis [21]. Horsman and Overgaard performed a meta-analysis which evaluated all clinical trials in which patients were randomized to receive radiation alone or radiation with hyperthermia. Twenty-three trials with 1861 patients were reviewed, and the odds ratio of locoregional control was in favour of radiation and hyperthermia (OR (95 % CI) = 1.80 (1.50–2.16)) for all tumour sites combined; in the five studies that included only head and neck cancer patients, the results suggest overall improvements when heating was added to radiotherapy (OR = 2.08 (1.28–3.39)) [16].

A controlled randomized study of interstitial hyperthermia and radiotherapy was conducted with the majority of the tumours located in the head and neck region [22]. The intended goal of hyperthermia in the study was to increase radiation sensitivity (maintain tumour temperature of 42.5 °C for 30 to 60 min); this regimen would not be expected to produce protein denaturation and necrosis associated with HIFU (55–90 °C for 20–30 s). In that study (which pre-dated the use of focused ultrasound techniques), there were no significant differences detected in treatment toxicity or tumour response and the authors concluded that ‘substantial technical improvements in heat delivery and dosimetry’ would be required for future studies [22]. Two other trials comparing radiation alone versus radiation and hyperthermia (42.5 °C for 30 to 60 min) in head and neck cancers found a benefit in overall survival [23, 24]. Tumour stage may have been an important factor influencing the response, as in one study, a survival benefit of adding heat was only seen in the stage III and IV patients [23], whereas in the other study where a clear benefit was seen, the patients were all stage IV [24]. Survival advantage with hyperthermia has been demonstrated for other tumour types such as the carcinoma of the cervix [25]. A prospective, randomized, multicenter trial investigating the effect of heat on the radiation response in tumours of the pelvic region reported that the benefit seen in the patients was primarily influenced by the large enhancement in the cervix group compared with tumours involving the rectum and bladder where no significant improvements were seen [26].

The majority of prior studies examining the use of hyperthermia with radiotherapy were completed using interstitial heating techniques, prior to the development of MR-guided focused ultrasound. With improvement in target delineation, real-time thermometry and heat dosimetry MRg-HIFU may provide more consistent and reproducible clinical benefits.

## Methods/Design: Hypothesis

MRg-HIFU of head and neck cancer with regional lymph node involvement is safe and feasible when performed prior to standard radiotherapy treatments. MRg-HIFU can cause de-vascularization and necrosis of a target lesion within the neck as demonstrated by post-treatment MRI.

## Primary objectives

The primary aim of this study is to assess safety, toxicity and feasibility of MRg-HIFU treatments to the head and neck region delivered prior to palliative radiotherapy.

## Secondary objectives

The secondary aim of this study is to assess changes caused by MRg-HIFU within the treated tumour regions based on post-treatment MRI.

## Inclusion criteria

- Age ≥18 years
- Able to give informed consent
- Weight <140 kg
- Biopsy-proven diagnosis of squamous cell or undifferentiated carcinoma of the head and neck region (skin and nasopharynx primarily included)
- Radiologic evidence of metastatic disease involving the lungs, liver or bone
- Radiologic evidence of neck lymphadenopathy with at least one target lesion measuring >3 cm in the largest dimension (recurrent or initial presentation)
- Assessed by the treating radiation/medical oncologists to undergo palliative radiotherapy and/or chemotherapy
- Target lesion visible by non-contrast MRI
- Target lesion accessible for MRg-HIFU procedure
- Able to communicate sensation during MRg-HIFU treatment

## Exclusion criteria

- Pregnant/nursing woman
- Unable to have contrast-enhanced MRI scan—standard institutional criteria
- Head and neck surgery (excluding biopsy) ≤6 weeks prior to study enrolment
- Chemotherapy or other systemic anti-cancer agents ≤6 weeks prior to enrolment
- Previous radiotherapy ≤6 weeks prior to enrolment
- Target lesion involves the skin surface causing ulceration, bleeding or discharge
- Target lesion circumferentially encompasses major blood vessels (carotid or jugular)
- Target lesion in contact with a hollow viscera
- Target lesion located in the skull, spine or mandible
- Fibrotic scar along a proposed HIFU beam path
- Orthopaedic implant along a proposed HIFU beam path or at site of target lesion
- Severe cardiovascular, neurological, renal or haematological chronic disease
- ECOG (Eastern Cooperative Oncology Group) performance status ≥3
- Active infection
- Unable to tolerate required stationary position during treatment
- Allergy to MRI contrast agent or sedation

## Preliminary ‘run-in’ study

A preliminary, run-in phase will be completed prior to initiating the main study. Five patients with newly diagnosed head and neck cancer will be recruited from the Head and Neck Oncology Programme at the study institution. These patients will have been fully staged with a previous MRI and undergo standard radiation and chemotherapy as per standard of care. Patients who volunteer and consent to participate will undergo simulation on the Philips MRI-HIFU system. Simulation will include adjusting patient position, immobilizing them on the MRI couch with a customized cushion and an MRI scan to obtain anatomic and temperature images. Patients will *not* receive any thermal treatment throughout this process. This will help determine the optimal patient position and ultrasound beam approach for different tumour locations. The information gathered from this run-in phase will aid the optimization of patient preparation and set-up and workflow for the ablative treatment.

## Study design

The main study is a prospective, pilot, single-centre, single-arm, non-randomized trial.

It is expected to take up to 12 months for a planned accrual of 10 patients in the study for MRg-HIFU treatments. Therefore, the entire study accrual period is anticipated to be up to 18 months (5 patients MRI only; 10 patients MRg-HIFU treatment).

### Recruitment

Patients will be recruited from the Head and Neck Oncology Programme at the study institution. Patients meeting the above inclusion and exclusion criteria will be informed about the study by their attending physician. Interested patients will meet with a research assistant and will be given a consent form and have their questions answered. Patients wishing to participate will sign the consent form and proceed to baseline data collection.

### Baseline data collection

**Fig. 1:**
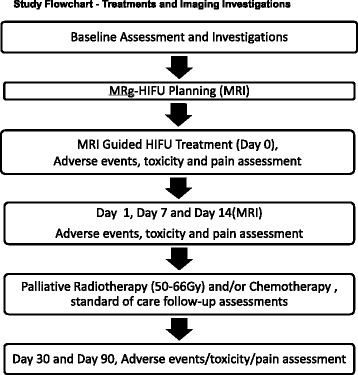
The study flowchart of treatments and imaging investigations provides an overview of the sequence of MRg-HIFU, imaging and follow-up during the study

A research assistant will collect standard baseline demographic information from participating patients, as well as help the patient to fill out the pain questionnaire, and record total analgesic consumption during the previous 24 h (see Fig. 1 and Table 1).

**Table 1:** Study investigations, treatments and follow-up summarize the full schedule of patient visits, treatment, imaging and assessments of adverse events

	Recruitment/baseline investigation	MR-guided HIFU treatment	Post-HIFU day 1	Post-HIFU day 7	Post-HIFU day 14	Palliative XRT/chemo	Post-HIFU day 30	Post-HIFU day 90
Physical examination	✓	✓	✓	✓	✓	soc	✓	✓
Blood work	✓					soc		
MRI	✓	✓			✓	soc		
Toxicity/SAE		✓	✓	✓	✓	soc	✓	✓
Pain and analgesic form		✓	✓	✓	✓	soc	✓	✓

Toxicity and SAE consists of anticipated adverse events screening, NCI CTCAE v4.03 toxicity score and SAE evaluating form when adverse events meet SAE criteria. Pain and analgesic: assessment consists of a visual analogue scale (VAS) and HIFU-related pain and analgesic form

*soc* standard of care, visits and assessments will follow regular institutional practice. *XRT* radiotherapy treatment, *SAE* serious adverse experience

### Baseline blood work

If not already available, participating patients will also attend for baseline blood work (CBC, electrolytes, creatinine, INR, PTT) within 2 weeks prior to the MRg-HIFU procedure (see Table 1). If the standard institutional blood work requirements for MR imaging with sedation are not satisfied, the patient is ineligible and will exit the study.

### Baseline MRI examination

If not already available (≤4 weeks prior to enrolment), participating patients will undergo MR imaging with IV contrast of the region of the target lesion (see Fig. 1 and Table 1). The treating radiation oncologist and radiologist will identify and measure the target lesion. The position of the target in relation to the skin, bone, major blood vessels, nerves and spinal cord will also be evaluated. If the imaging-related inclusion and exclusion criteria are not satisfied, the patient is ineligible and will exit the study. If the criteria are satisfied, the patient is eligible and will proceed to the MRg- HIFU procedure.

### MR-guided HIFU device description/overview

Philips MRg-HIFU (Philips Sonalleve) offers the physician two methods for ablating tissue. A point to point method allows specific heating of cells drawn within the treatment area. HIFU ablation is performed with the US energy adjusted to achieve a tissue heating to 55–90 °C over a ~30-s time period. The source then moves to a different non-contiguous location, and HIFU ablation is repeated. Each individual ‘point’ represents an ablative region with an elliptical shape with an average diameter of 4.0 mm and length of 8.1 mm producing a volume of approximately 0.067 cc [27]. The treatment is delivered point-by-point until the larger target has been encompassed. The second method, a volumetric plan, also allows heating of cells within a treatment area. The difference is that the transducer applies heat in a continuous manner to adjacent points in a treatment area. Heating by this method makes it possible to raise the temperature not just by ablating point per point but also as a controlled thermal dose over significantly larger volumes. Volume treatment versus point-by-point substantially reduces treatment time, allowing for complete target coverage and delivery of optimal thermal dose. (For this pilot study, a point-by-point method will be used to treat the target lesion.)

The MRg-HIFU system displays real-time temperature data overlaid on proton or T2 anatomical images, accumulated thermal dose information in the treated area and an the ultrasound therapy parameters such as transmitted output power that is used to generate the thermal dose. Additionally, the operator can use a mouse to select specific thermal areas to obtain temperature readout. The display is updated every 1–2 s with new information.

The system also offers visual and audible alerts to inform the operator when an area exceeds the user-defined critical values. Available user alerts are when:

1. Temperature elevations occur at critical values, i.e. at 40 °C, in user-defined warning zone areas
2. A significant drop in transmitted power (10 %) occurs, or
3. A significant drop in temperature versus time curve at the focus occurs
4. The cavitation detector reaches a critical level—this device detects when input ultrasound energy creates bubble activity and not direct temperature elevation

The ultrasound system will automatically shutdown if:

1. A bubble activity is detected in the US transmission path
2. The transducer overheats
3. Measured output power is higher or substantially lower than the requested power
4. High levels of reflected power are detected in the amplifiers
5. Any system control element fails to respond correctly to a control request

A system shutdown is indicated both by the UI and by an indicator light on the operator console. After a safety shutdown or use of a stop button by the patient, a manual reset or another clear intervention by the operator is required before use of the ultrasound system can be continued.

### MR-guided HIFU procedure

On the procedure day, an IV catheter will be inserted to deliver MR contrast media and medications such as sedation and analgesics as per standard operating procedures within the MR room (see Fig. 1 and Table 1). In the MR room, the patients will be asked to lie on the HIFU patient table inside the MRI magnet. The treating radiation oncologist and MRg-HIFU operator will locate the target tissue and mark the volume to be treated using MRI images.

The operator starts the treatment and monitors the progress of the treatment with MR thermal and dose maps to ensure adherence to the treatment plan. Philips MRg-HIFU (Philips Sonalleve) will be used to ablate the targeted tumour tissue. A point to point treatment method will be employed in order to achieve target tissue heating to between 55 and 90 °C over a period of up to 30 s. The source then moves to a different non-contiguous location, and HIFU ablation is repeated. This is done point-by-point until all the points have been treated. For this initial research study, a margin of 1 cm between the region of ablation and the adjacent critical structures will be adhered to. Therefore, the peripheral margins of the tumour may be untreated. When the whole planned volume has been treated, the operator stores the full treatment history. The patients are then conducted to the recovery room for medical supervision. Before discharge, follow-up instructions will be given to the patient. The same MRI device will be used on day 14 after HIFU to perform a standard, contrast-enhanced, follow-up MRI scan of the head and neck to assess for changes in the target lesion and the neck, such as decreased enhancement or necrosis.

### Palliative radiotherapy/chemotherapy

All patients in the study will undergo palliative radiotherapy and/or chemotherapy. Treatments will begin at least 14 days after the MRg-HIFU treatment. Palliative radiotherapy treatment will be administered to the pre-treatment target lesion and in addition may encompass other tumour regions of the head and neck. The prescribed dosage for patients who have not received previous radiotherapy will be between 50 and 66 Gy over 4–7 weeks; in previously irradiated patients, the dose will be determined at the discretion of the treating radiation oncologist.

### Safety and toxicity assessment

The patient will be assessed by a physician investigator and clinical research assistant (CRA) on the MRg-HIFU treatment date, then 1 day, 1 week, 2 weeks, 1 month and 3 months afterward. The first and third month visits may coincide with other palliative treatments that occur after the MRg-HIFU therapy.

At each visit, including the treatment date, the Anticipated Adverse Effects Screening Assessment and a Pain/Analgesics form will be completed by the CRA and physician. If specific adverse effects are identified, then the National Cancer Institute Common Toxicity Criteria (NCI CTCAE 4.03) will be utilized to assign a grade/score to the severity of the effect. At any follow-up visit or any other time within 60 days following the MRg-HIFU treatment, serious adverse experiences (SAE) as defined in the SAE form will be evaluated by a physician and documented. In addition, any SAE, including death due to any cause, which occurs to any patient who entered into this study up to 60 days following the MRg-HIFU treatment, will be reported within 24 h to Philips Healthcare (regulatory sponsor), necessary regulatory agencies, research ethics boards and fellow investigators. If any patient develops a SAE or grade 4 toxicity, then no further patient will be treated in the study until the event has been reviewed by the investigators and deemed unlikely to be related to the study treatment or unlikely to recur in future patients.

### Feasibility

Feasibility of the procedure will be assessed and recorded by the following:

- Number of patients who meet clinical eligibility criteria and are enrolled on study.
- Number of patients who complete the first planning MRI/number deemed to have an appropriate target lesion within the neck to proceed to MRg-HIFU.
- Number of patients who attend treatment/completing the MRg-HIFU ablation session.
- If patients are unable to complete the treatment, the specific reason will be recorded: e.g. pain associated with procedure, physical set-up of transducer-skin coupling, organ motion, critical structure constraints, and hyperthermia delivered instead of HIFU.
- Actual treatment delivered vs. MRI plan: number of sonications, volume receiving ablative temperature, formation of any unintended lesions outside of the planned volume.
- Imaging evidence of anticipated HIFU effects: pre-treatment MRI parameters compared with post-treatment day 1 and day 14 within the target region will assess for decrease in contrast enhancement (de-vascularization) and increased T2 signal intensity (necrosis).

## Unplanned exit from the study

In addition to the criteria for exit mentioned above, patients may choose to exit the study at any time.

## Statistical methods

Demographic data will be organized with descriptive statistics. All serious adverse events will be recorded and reported as described above. Toxicity and feasibility measures will be summarized for all patients. Given the small number of patients in this pilot study, no inferential statistical analysis is planned.

## Sample size

The study plans to accrue 10 patients. (An additional of five patients will have MRI only.) This number is typical for a phase 1 pilot study in patients with advanced cancer to establish safety and feasibility. Based on the expected number of eligible patients referred to the Head and Neck Oncology Programme at the study institution, this number also seems a reasonable target (>25 eligible patients per year). No previous studies of HIFU for head and neck cancer are available for direct assessment of patient risk. The risk of serious adverse events resulting from the MRg-HIFU is estimated to be low to moderate as the technology is being used safely for other interventions within other body sites with safe outcomes.

## Discussion: Implications of the study

If proven safe, feasible and efficacious, this pilot study may allow the investigators to proceed with studies involving larger numbers of patients who have potentially curable disease. Future studies may examine the use of MRg-HIFU to enhance standard chemoradiotherapy treatments of locally advanced, unresectable head and neck tumours. Potential future applications in the head and neck region include hyperthermia radiosensitization, combined modality tumour ablation and thermally activated chemotherapy.
